# Identification of Putative Virulence Genes by DNA Methylation Studies in the Cereal Pathogen *Fusarium graminearum*

**DOI:** 10.3390/cells10051192

**Published:** 2021-05-13

**Authors:** Francesco Tini, Giovanni Beccari, Gianpiero Marconi, Andrea Porceddu, Micheal Sulyok, Donald M. Gardiner, Emidio Albertini, Lorenzo Covarelli

**Affiliations:** 1 Department of Agricultural, Food and Environmental Sciences, University of Perugia, Borgo XX Giugno, 74, 06121 Perugia, Italy; francensco.tini@collaboratori.unipg.it (F.T.); giovanni.beccari@unipg.it (G.B.); emidio.albertini@unipg.it (E.A.); lorenzo.covarelli@unipg.it (L.C.); 2 Department of Agriculture, University of Sassari, Viale Italia, 39a, 07100 Sassari, Italy; aporceddu@uniss.it; 3 Department of Agrobiotechnology (IFA-Tulln), University of Natural Resources and Applied Life Sciences, Vienna (BOKU), Konrad Lorenz Strasse, 20, A-3430 Tulln, Austria; michael.sulyok@boku.ac.at; 4 Commonwealth Scientific and Industrial Research Organisation, Agriculture and Food, 306 Carmody Road, St Lucia, QLD 4067, Australia; donald.gardiner@csiro.au

**Keywords:** DNA methylation, *Fusarium graminearum*, *in vitro* subcultures, virulence reduction, ddRAD-MCSeEd, virulence genes

## Abstract

DNA methylation mediates organisms’ adaptations to environmental changes in a wide range of species. We investigated if a such a strategy is also adopted by *Fusarium graminearum* in regulating virulence toward its natural hosts. A virulent strain of this fungus was consecutively sub-cultured for 50 times (once a week) on potato dextrose agar. To assess the effect of subculturing on virulence, wheat seedlings and heads (cv. A416) were inoculated with subcultures (SC) 1, 23, and 50. SC50 was also used to re-infect (three times) wheat heads (SC50×3) to restore virulence. *In vitro* conidia production, colonies growth and secondary metabolites production were also determined for SC1, SC23, SC50, and SC50×3. Seedling stem base and head assays revealed a virulence decline of all subcultures, whereas virulence was restored in SC50×3. The same trend was observed in conidia production. The DNA isolated from SC50 and SC50×3 was subject to a methylation content-sensitive enzyme and double-digest, restriction-site-associated DNA technique (ddRAD-MCSeEd). DNA methylation analysis indicated 1024 genes, whose methylation levels changed in response to the inoculation on a healthy host after subculturing. Several of these genes are already known to be involved in virulence by functional analysis. These results demonstrate that the physiological shifts following sub-culturing have an impact on genomic DNA methylation levels and suggest that the ddRAD-MCSeEd approach can be an important tool for detecting genes potentially related to fungal virulence.

## 1. Introduction

Fusarium Head Blight (FHB) is one of the most widespread and damaging diseases of cereal crops, such as bread and durum wheat and barley, capable of strongly impairing not only yield but also quality, by contaminating grains with mycotoxins. The disease is caused by several members of the *Fusarium* species complex [1]. *F. graminearum* is globally considered the most dangerous FHB pathogen due to its aggressiveness and diffusion worldwide [2,3,4]. The pathogen is able to biosynthesize mycotoxins belonging to the type B trichothecenes, such as deoxynivalenol (DON) and nivalenol (NIV) [5] as well as the DON acetylated forms: 3-acetyl-deoxynivalenol (3-ADON) and 15-acetyl-deoxynivalenol (15-ADON) [4,6].

All living organisms can adopt strategies to enable rapid adaptation to new environmental conditions, without changing the DNA sequence [7,8]. For example, to respond to the environmental changes or biotic and abiotic stresses, some organisms adjust physiological and development machinery by gene expression regulation. DNA methylation and demethylation of cytosine play key roles in such a strategy [9,10,11,12]. Cytosine methylation is conventionally classified in CG, CHG, and CHH sequence contexts, where H is adenine, cytosine, or thymine. DNA methylation involves the addition of a methyl group to cytosine to produce 5-methylcytosine (5mC). Furthermore, the addition of the same group to adenine has been recently explored (N6-methyladenine, 6 mA), [13,14]. Methylation changes on cytosine residues are important for transposon silencing, epigenetic regulation, and genome expression [15,16,17]. Generally, methylation is related to the silencing of genes and transposable elements, whereas demethylation is correlated to active transcription [18], even if the reverse has also been described [15]. DNA methylation is catalysed by a conserved set of proteins called DNA methyltransferases (MTases) [19]. DNA MTases in eukaryotes belong to five different groups based on their structure and functions [20,21,22]. DNA MTase homologs have been identified in many fungal pathogens, including *F. graminearum* [23]. In pioneering studies, the histone proteins DIM-2, DIM-5, and HP1 were defined to be essential for DNA methylation in *Neurospora* [24,25,26]. Homologues for these three proteins are present in all sequenced *Fusarium* species [27], demonstrating that the DNA methylation machinery is present in this genus [28].

Some pathogenic fungi may partially lose or attenuate their virulence in response to environmental changes [29] as well as in response to other external factors, such as prolonged subculturing on artificial rich media [30]. Virulence may be restored if the attenuated strains are re-inoculated onto healthy host tissues. In the present work, we analysed whether virulence changes due to subculturing in different media/hosts was associated with DNA methylation changes and if these changes affected genes known to be involved in virulence regulation toward different hosts.

The loss of aggressiveness, following subculturing in artificial rich media, was evaluated by (i) execution of *in planta* virulence assays, (ii) characterization of secondary metabolites biosynthesis, and (iii) determination of *in vitro* fungal development and conidiation. Furthermore, the colony that had undergone consecutive transfers for one year on an artificial, rich, nutrient medium was used to infect healthy bread wheat heads. The DNA extracted from the last *in vitro* subculture was compared to the DNA isolated from mycelia sampled on infected wheat heads. Several genes affected by methylation level changes have already been demonstrated to be involved in virulence toward the host.

## 2. Materials and Methods

### 2.1. Fungal Strain and Subculturing

**Figure 1:**
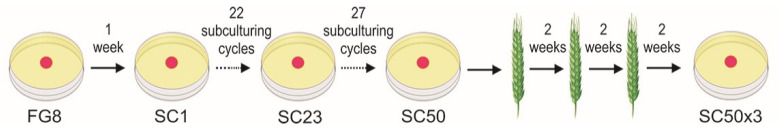
Experimental design followed throughout the experiment.

*F. graminearum* strain FG8 (15-ADON producer) from the fungal collection of the Department of Agricultural, Food, and Environmental Sciences (University of Perugia, Perugia, Italy) was used for all experiments. FG8 was isolated from durum wheat grain, molecularly identified and characterized for the *in vitro* mycotoxigenic profile [31]. The experimental design is shown in Figure 1.

To prepare the subcultures’ inoculum, FG8 was cultured on potato dextrose agar (PDA, Biolife Italiana, Milan, Italy) for 50 weeks. Briefly, a piece of fungal mycelium was cultured on PDA in a 9-cm Petri dish at 22 °C. After one week, one mycelium plug (0.5 cm of diameter) was used to inoculate a sterile PDA plate that was incubated for another week at 22 °C, whereas the rest of the mycelium was cut in small pieces and stored into 2-mL plastic tubes (Eppendorf, Hamburg, Germany) at −80 °C and represented the SC1 inoculum. The same actions were repeated every week for 50 weeks, obtaining a total of 50 subcultures stored at −80 °C (from SC1 to SC50). Sterile PDA plates were inoculated with mycelium plugs deriving from SC1, SC23, and SC50 samples for further analyses.

SC50×3 subcultures were obtained from sterile PDA plates inoculated with mycelium derived from three head-to-head passages, as described in Section 2.2.2.

### 2.2. Virulence Assays

#### 2.2.1. Crown Rot Assay

The virulence assay on the stem base of bread wheat was carried out following the method previously described [32,33,34]. The mycelium of SC1, SC23, and SC50 was cut in small squares and homogenised with 12 mL of sterile water with a Mixer Mill MM400 (Retsch, Haan, Germany) to obtain a gel for pipetting. Bread wheat seeds (cv. A416, an Italian cultivar with well-known susceptibility to FHB) were previously surface sterilized with a solution composed of 7% sodium hypochlorite (8% *v/v*), 98% ethanol (10% *v/v*), and sterile, deionised water (82% *v/v*) for 5 min and rinsed three times with sterile, deionised water. Surface-sterilized seeds were sown in 6 × 8 × 8 cm pots (10 seeds per pot), and filled with a sterile soil mix (50% sand and 50% peat). Pots were incubated at 22 °C with a 15/9 h day/night light cycle. A 3-cm-long PVC collar (3-mm internal diameter) was placed around the emerging coleoptiles. When the second leaf was fully expanded, plants were inoculated by injecting 700 µL of inoculum into the space between seedling and the PVC collar. PDA macerated with sterile water was used as a control treatment. Three replicates (corresponding to three different pots, 10 plants per pot/replicate) for each FG8 subculture and for the control were realized for a total of 12 pots (120 plants). The inoculated seedlings were covered by plastic bags for 3 days to keep the moisture high. Seedlings were maintained for 25 days at 22 °C with a 15/9 h day/night light cycle.

Stem-base infections were evaluated by measuring the length (cm) of the necrotic area on the first leaf and the presence/severity of necrosis across leaf sheaths with a 0–17 arbitrary scale (clean = 0; coleoptile = 1–2; 1st leaf = 3–4–5; 2nd leaf = 6–7–8; 3rd leaf = 9–10–11; 4th = 12–13–14; 5th leaf = 15–16–17).

The fungal subcultures virulence toward the bread wheat stem base was evaluated using crown rot disease index (DI). DI was calculated as the product between the average length of the necrotic area on the first leaf (cm) and the average value (0–17) of necrosis across leaf sheaths of 10 plants for 3 replicates.

#### 2.2.2. Fusarium Head Blight Assay

Flasks containing 300 mL of mung bean broth were inoculated with a SC50 mycelium plug. Mung bean broth was prepared by boiling 1 L of sterile water and adding 40 g of mung beans for 10 min. Subsequently, beans were removed from the broth by filtering with cheesecloth and the broth was autoclaved. The inoculated flasks were shaken on an orbital shaker at 150 rpm for 10 days at room temperature and 12/12 h light/dark. The fungal broth was filtered through Miracloth (Millipore Corporation Billerica, MA, USA) and the conidia suspension concentration was adjusted to 1 × 10^6^ conidia mL^−1^ using a haemocytometer to count the cells. Sterilised seeds were incubated in Petri dishes for one day in the dark at 4 °C on water-soaked filter paper and three days in the dark at room temperature for germination. Germinated seeds were transplanted into 9 × 9 × 13 cm pots (one seed per pot) filled with peat and placed in a growth chamber at 23 °C with a photoperiod of 16 h. At mid-anthesis, wheat heads were point inoculated with macroconidia of SC50 by pipetting 10 µL of conidial suspension, containing approximately 10^4^ conidia. The inoculum was injected between the glumes of a central spikelet. Heads were covered in plastic bags for 7 days to increase moisture content. Two weeks after inoculation, a little piece of mycelium was scraped from inoculated heads and used for inoculating other healthy wheat heads. This was repeated for three consecutive direct head-to-head transfers to obtain a sample named SC50×3. A portion of scraped mycelium of SC50×3 was cultured on PDA and stored at −80 °C for DNA extraction or used to inoculate mung bean flasks to obtain SC50×3 conidia inoculum, as described for the FHB assay. At mid-anthesis, heads were point inoculated as mentioned above, with 10 µL of conidial suspension containing approximately 10^4^ conidia. A total of 15 heads per subculture were inoculated (5 heads per replicate) for a total of 75 heads, including control (sterile-water inoculation). After inoculation, heads were covered for 72 h in plastic bags to maintain a high humidity level and promote the infection. Inoculated plants were placed into a growth chamber with a photoperiod of 16 h at 23 °C. Symptoms caused by the different subcultures were assessed at 14 days post-inoculation (dpi), determining the proportion of spikelets of each head that displays browning symptoms.

### 2.3. In Vitro Growth Rate Assay

One mycelium plug of 0.5 cm diameter of SC1, SC23, and SC50 was taken from the edge of a 4-day-old colony and placed in the middle of the plates containing PDA. Six replicates per subculture were realized for a total of 18 plates. The growth rate was evaluated measuring the mycelial diameters in the two perpendicular directions, as previously described [35]. The diameters of the colonies were measured alongside the two axes. The growth value was calculated as the average of the measures taken from the two axes for three replicates per subculture and expressed in centimeters.

### 2.4. In Vitro Conidial Production

Three PDA Petri dishes per sample (three replicates) were inoculated with one mycelium plug of each subculture (diameter of 0.5 cm) and incubated for 4 weeks at room temperature, under near-UV light for 12 h per day. At the end of the incubation period, 15 mL of sterile water were added with a pipette to each incubated plate and the mycelium was scraped and mixed to the added water with a sterile spatula. The conidia were separated from the mycelium by Miracloth filtration. Conidia concentration was estimated with a haemocytometer and conidia production was calculated as the average of three replicates per sample.

### 2.5. Determination of Secondary Metabolites Biosynthesized In Vitro by F. graminearum Subcultures

#### 2.5.1. *F. graminearum* Subcultures Preparation

Ten milliliters of deionized, sterile water were added to 20 g of rice kernels and placed into 100-mL glass flasks, autoclaved three times on alternate days, and inoculated with one mycelium plug per sample. Flasks were incubated for 4 weeks at 22 °C in the dark and the developed cultures were milled with mortar and pestle and stored at −80 °C. Three replicates per sample were realized. Three non-inoculated flasks with rice kernels were used as controls.

#### 2.5.2. Extraction and Analysis of Secondary Metabolites

Five grams of each ground sample were extracted using 20 mL of extraction solvent (acetonitrile-water-acetic acid, 79:20:1, *v/v/v*) followed by a 1 + 1 dilution using acetonitrile-water-acetic acid (20:79:1, *v/v/v*) and direct injection of 5 μL of diluted extract. Concentrations exceeding the linear range of the detector were quantified by reanalysis of the extracts after further dilution steps (1:50 and 1:1000, respectively). LC-MS/MS screening of target fungal metabolites was performed with a QTrap 5500 LC-MS/MS System (Applied Biosystems, Foster City, CA, USA) equipped with a Turbo Ion Spray electrospray ionization (ESI) source and a 1290 Series HPLC System (Agilent, Waldbronn, Germany). Chromatographic separation was performed at 25 °C on a Gemini^®^ C18-column, 150 × 4.6 mm i.d., 5-μm particle size, equipped with a C18 4 × 3 mm i.d. security guard cartridge (all from Phenomenex, Torrance, CA, USA). The chromatographic method as well as chromatographic and mass spectrometric parameters are described in Reference [36]. Confirmation of positive analyte identification was obtained by the acquisition of two MRMs per analyte (with the exception of MON and three nitro propionic acids that exhibit only one fragment ion), which yielded 4.0 identification points according to the commission decision (Commission Decision, 2002). In addition, the liquid chromatography retention time and the intensity ratio of the two MRM transitions agreed with the related values of an authentic standard within 0.03 min and 30% rel., respectively. Quantification was performed via external calibration using serial dilutions of a multi-analyte stock solution. Results were corrected for apparent recoveries obtained for wheat [36]. The accuracy of the method is verified on a continuous basis by regular participation in proficiency testing schemes.

### 2.6. DNA Extraction

To proceed with methylation analysis, DNA from SC50 and SC50×3 was extracted. In detail, SC50 was grown in Petri dishes containing PDA. Fungal mycelium was scraped using a spatula, and placed into 2-mL sterile plastic tubes with a steel bead at −80 °C. The SC50×3 mycelium developed on host tissues after three head-to-head consecutive transfers (Section 2.2.2) was collected with tweezers and stored in 2-mL plastic tubes with one steel bead at −80 °C. Mycelium samples (SC50 and SC50×3) were freeze-dried (Heto Power Dry LL3000) and reduced to a fine powder whit using a Mixer Mill MM400 (Retsch). Genomic DNA of the four samples was obtained using a PureLink™ Plant Total DNA Purification Kit (Thermo Fisher Scientific, Walthman, MA, USA) according to the manufacturer’s instruction. Extracted DNA concentration was quantified with a Qubit^®^ 3.0 Fluorometer (Thermo Fisher Scientific), using a dsDNA High Sensitivity (HS) Assay (Thermo Fisher Scientific) kit, following the manufacturer’s protocol.

### 2.7. DNA Methylation Analysis

The library set-up protocol was performed according to Reference [37]. Three specific enzyme combinations were chosen to infer the CG (*Aci*I/MseI), CHG (*Sex*AI/*Mse*I), and CHH (*Eco*T22I/*Mse*I), methylation contexts, respectively. Briefly, for each library, 150 ng DNA were double-digested with one of these enzyme combinations following the protocol previously described [37,38]. The libraries were then pooled, purified using magnetic beads (Agencourt AMPure XP, Beckman Coulter, MA, USA), size selected by gel electrophoresis, and purified using QIAquick Gel Extraction kits (Qiagen, Hilden, Germany) for fragments ranging from 250 bp to 600 bp. Size-selected libraries were quantified using a Qubit^®^ 3.0 Fluorometer (Thermo Fisher Scientific), and a normalized DNA amount (15 ng) was amplified with a primer that introduced an Illumina index (at the Y common adapter site) for demultiplexing. Following PCR with uniquely indexed primers, multiple samples were pooled and subjected to PCR-enrichment, as previously described [37]. The grouped libraries were pooled in an equimolar fashion, and the final library was Illumina-sequenced using 150-bp single-end chemistry. Raw reads from the Illumina sequencing of the CG, CHG, and CHH libraries were analyzed following the protocol and the pipeline previously described [37].

The relative methylation levels at each site were calculated following a described procedure [37] and the DMPs (Differentially Methylated Positions) were called following the methyl kit’s manual best practices [39]. The mapping of the DMPs in the same scaffold and as closer than a given threshold provided their clustering together to identify the DMRs (Differentially Methylated Regions), as previously reported [37].

#### Synteny Block and Statistical Analysis

Synteny block analysis was performed with MCSCANX with default settings. *F. graminearum* proteins were used as a query against a database of *F. verticillioides* proteins for BLASTP homology searches. The BLASTP results were exported in a tabular format (m 8). The criteria for synteny block analysis were: match score 50, match size >5, gap_penalty of −1, and max gaps of 25. The chromosomes of *F. graminearum* were partitioned in an adjacent window of 20 kb and, for each of these regions, the proportion of mapping genes collinear to *F. verticillioides* was calculated. Chromosomal windows with a portion of collinear genes below 50% were considered to be non-conserved. The relative abundance of DMPs and DMR mapping in conserved and not conserved (NC) regions were compared with a permutation test.

The effect of subculturing on pathogen virulence, mycelium growth, conidia production, and metabolite biosynthesis were tested by one-way ANOVA and Duncan’s multiple comparison tests, as implemented in the program DSAASTAT [40].

## 3. Results

### 3.1. Subculturing Reduces Fungal Virulence, but Passaging Can Rescue These Defects

To assess the effect of continuous subculturing on virulence, three subcultures of the FG8 strain (SC1, SC23, and SC50) were selected for virulence assays toward bread wheat with inoculations performed on stem bases.

**Figure 2:**
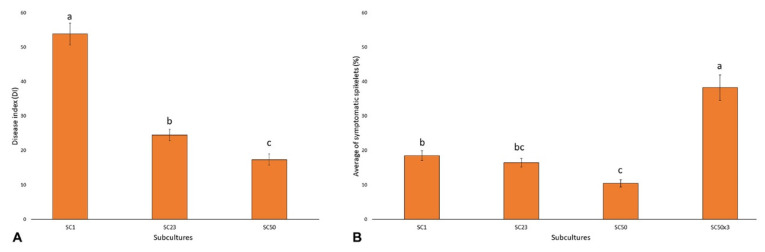
(**A**) Stem base Disease Index (DI) of soft wheat inoculated with subcultures SC1, SC23, and SC50 of *F. graminearum* strain FG8. Columns represent the average of three replicates (± SE) with each composed of 10 plants. Values with different letters are significantly different based on Duncan’s multiple comparison tests (*p* < 0.05). (**B**) Average percentage of symptomatic spikelets (%) of heads point-inoculated with the FG8 different subcultures. Columns represent the average of three replicates (±SE), in which each is composed of five heads. Values with different letters are significantly different based on Duncan’s multiple comparison tests (*p* < 0.05).

A different aggressiveness between SC1, SC23, and SC50 was observed (Figure 2A). All three subcultures showed the ability to cause the typical necrotic lesions on the first leaves and across leaf sheaths of soft wheat plants. In detail, crown rot disease index (DI), calculated as the product between the average length of the necrotic area on the first leaf (cm) and the average value (0–17), was 53.8, 24.5, and 17.4 in plants inoculated with SC1, SC23, and SC50, respectively. The DI decrease was significant (*p* < 0.05) and followed the gradient SC1 > SC23 > SC50.

The aggressiveness of the same subcultures was also evaluated toward bread wheat heads. All three subcultures were able to induce the typical FHB bleached spikelets with aggressiveness decreasing by the subculturing time (Figure 2B). These subcultures were compared with SC50×3, which was obtained from mycelia derived from three head-to-head passages, as described in MM. The aggressive average levels described a trend with SC50×3 > SC1 ≥ SC23 ≥ SC50 (Figure 2B). In detail, the initial subculture (SC1) caused 18.5% of symptomatic spikelets whereas the virulence of SC50 was significantly (*p* < 0.05) reduced when compared to the first one, with only 10% of spikelets showing symptoms. SC23, with an average of 16% infected spikelets, showed an intermediate aggressiveness in comparison to SC1 and SC50. These results showed that continuous subculturing on PDA caused a pathogen virulence decrease as a consequence of its adaptation to a nutrient-rich medium while three transfers of SC50 on wheat heads fully restored virulence (38% of bleached spikelets) to a degree even higher than that observed for SC1 (*p* < 0.05).

### 3.2. Subculturing Affects Conidial Production but Not an In Vitro Growth Rate

**Figure 3:**
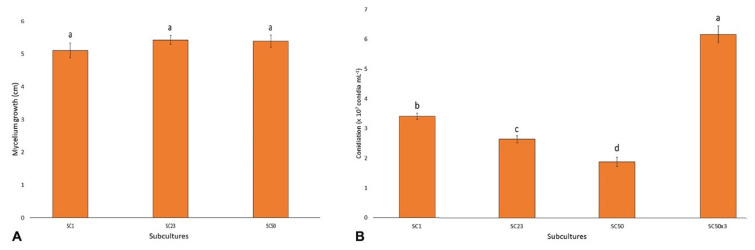
(**A**) Subcultures development (cm) along the diameters of Petri dishes containing PDA. Columns represent the average of three replicates (±SE). Values with the same letters are not significantly different (*p* > 0.05) based on Duncan’s multiple comparison tests. (**B**) Average conidia production by SC1, SC23, SC50, and SC50×3 developed on PDA under near-UV light for 28 days. Columns represent the average of three replicates (±SE). Values with different letters are significantly different based on Duncan’s multiple comparison tests (*p* < 0.05).

Mycelium growth rates from subcultures SC1, SC23, and SC50 were measured on PDA plates. Despite the three subcultures having different adaptation times on PDA (1, 23, or 50 weeks), no significant differences in growth rate were observed (*p* = 0.42) after 5 days (Figure 3A). In detail, SC1 showed an average growth of 5.1 cm along the axes, whereas SC23 and SC50 showed an average growth of 5.4 cm.

Conversely, significantly different conidia productions by the varying subcultures were detected (Figure 3B). In detail, conidiation followed the significant (*p* < 0.05) gradient: SC50×3 > SC1 > SC23 > SC50. In addition, after *in vivo* passages of the last subculture (SC50) for three times onto wheat head tissues, this strain produced a high number of conidia, which resulted in an even higher number (*p* < 0.05) than that observed in the first subculture (SC1).

### 3.3. Subculturing Does Not Affect In Vitro Secondary Metabolite Production

The accumulation of the main secondary metabolites detected in SC1, SC23, SC50, and SC50×3 subcultures are reported in Table S1.

In general, no significant differences in secondary metabolite biosynthesis between the four subcultures were detected. In detail, SC1 produced 19,900 μg kg^−1^ of 15-ADON in addition to 36,600 μg kg^−1^ of DON. A very low production of 3-ADON (700 μg kg^−1^), NIV (114 μg kg^−1^), and sambucinol (412 μg kg^−1^) was also detected. In addition, SC1 biosynthesised 56,000 μg kg^−1^ of zearalenone (ZEN) and some ZEN derivatives, such as 1700 μg kg^−1^ of alpha-zearalenol and 12,000 μg kg^−1^ of beta-zearalenol. Other metabolites such as culmorin (3400 μg kg^−1^), 15-hydroxyculmorin (4200 μg kg^−1^), and fusarin C (54,000 μg kg^−1^) completed the SC1 *in vitro* mycotoxigenic profile. All the reported values are the average of three replicates. As mentioned before, the subculturing process performed on PDA as well as the following passages on the host head tissues did not significantly alter the ability of the fungus to produce secondary metabolites. For example, the production of DON showed an increase (even if without significant differences, *p* > 0.05) going from SC1 to SC23 (60,000 μg kg^−1^) and to SC50 (74,000 μg kg^−1^). A partial, non-significant decrease was detected for SC50×3 (53,000 μg kg^−1^).

The 15-ADON biosynthesis levels did not show significant changes between subcultures (*p* = 0.91). A very similar amount was detected in all subcultures (SC1 = 19,900 μg kg^−1^, SC23 = 16,600 μg kg^−1^, SC50 = 18,100 μg kg^−1^, SC50×3 = 19,400 μg kg^−1^).

Similarly, 3-ADON biosynthesis was nearly the same in all subcultures (*p* = 0.37). In detail, 1100 μg kg^−1^ of 3-ADON were produced by SC23 and 1400 μg kg^−1^ by SC50 and SC50×3. NIV and sambucinol were detected with similar levels for SC1, SC23, SC50, and SC50×3 (*p* = 0.81 and *p* = 0.70, respectively). Culmorin levels showed a decreasing trend that was followed by a restoration after host re-infection, whereas 15-hydroxiculmorin increased from SC1 to SC50 and then decreased again on SC50×3, but both without significant differences (*p* = 0.96 for culmorin and *p* = 0.57 for 15-hydroxiculmorin).

Subculturing induced a very small increase of ZEN production while plant re-infection brought it back to the SC1 levels, even if no significant differences were detected (*p* = 0.32). In addition, alpha-zearelenol and beta-zearalenol did not show any significant differences (*p* = 0.87 and *p* = 0.57). Among other *Fusarium* metabolites, fusarin C showed a decrease in the total amount produced following the three passages but, again, without significant differences (*p* = 0.34).

Finally, no significant variations were detected for butenolid (*p* = 0.59), gibepyron D (*p* = 0.22), aurofusarin (*p* = 0.60), and rubrofusarin (*p* = 0.24) biosynthesis among the different subcultures.

### 3.4. DNA Methylation Analysis

#### 3.4.1. Identification of Differentially Methylated Positions and Differentially Methylated Regions

MCSeEd (Methylation Context Sensitive Enzyme ddRAD) [37,38] was used to investigate DNA methylation changes induced by subculturing. To this end, next-generation sequencing (NGS) libraries from genomic DNA purified from PDA plates (SC50) and wheat heads (SC50×3) were constructed. Therefore, a total of 12 libraries were produced by double restriction ligations with each using *Mse*I in combination with one of the three methylation-sensitive enzymes *Aci*I, *Sex*AI, and *Eco*T22I, for the CG, CHG, and CHH contexts, respectively (Supplementary Table S2).

After quality control, a mean of 822,679 thousand 150-bp-long reads from each library were obtained and aligned to the *F. graminearum* PH-1 reference genome [41]. Only reads mapped at unique genomic positions were retained. Thus, considering the three different contexts, a total of 2,899,673, 4,755,848, and 1,516,029 reads were mapped uniquely on the reference genome (92.6% of the total reads, with a minimum of 86.8% for *Aci*I, and a maximum of 96.6% for *Sex*AI) and were classified as MCSeEd loci (Supplementary Table S3).

Therefore, a total of 138,119 loci containing cytosines (120,439 in symmetric, and 17,680 in asymmetric contexts) (Supplementary Table S4) were identified.

The mapping location of each MCSeEd locus was investigated to determine whether it fell within a gene window that included the region within 0.5 kb upstream of the transcription start site (TSS), the transcribed region (i.e., the gene body), and the region within 0.5 kb downstream of the transcription termination site (TTS). Furthermore, 92% (*Aci*I), 91% (*Sex*AI), and 87% (*Eco*T22I) of the identified MCSeEd loci were included within these gene windows (Supplementary Figure S1).

After normalization of the MCSeEd loci, the sites covered by a total number of reads <4 or showing excessive read-count variation among the replicates (standard deviation of 5 for CG and 10 for GHG and CHH) were discarded. The remaining sites were used to estimate a total of 13,899 DMPs, out of the 138,119 MCSeEd loci, with significantly altered methylation levels between the SC50×3 and SC50 samples (false discovery rate, ≤0.05). Of these, 12,326 belonged to symmetric contexts, and 1573 belonged to asymmetric contexts (Supplementary Table S4).

Principal component analysis was used to graphically portray the samples based on the DMPs’ methylation levels (Supplementary Figure S2).

The first latent component (PC1) accounted for 71.6%, 78.4%, and 71.8% of the total variance for the CG, CHG, and CHH, contexts, respectively, and clearly discriminated between SC50×3 and SC50, indicating that the head-to-head transfer of mycelium induced genome-wide methylation changes. Accordingly, complete linkage clustering of the methylation levels at DMPs clearly separated the SC50×3 and SC50 (Supplementary Figure S2).

Considering all of the methylation changes being induced by host colonization in the replicates, we observed 1.4-fold (CG) to 1.15-fold (CHH) more methylation decreases than increases in response to healthy heads’ infection whereas, for CHG, the proportion of methylation changes was 1.12 in the other direction.

Genomic regions with co-regulated methylation changes upon subculturing, known as DMRs, were identified. In total, 932 DMRs were scored for CG (874), CHG (19), and CHH (39) contexts (Supplementary Table S5).

**Figure 4:**
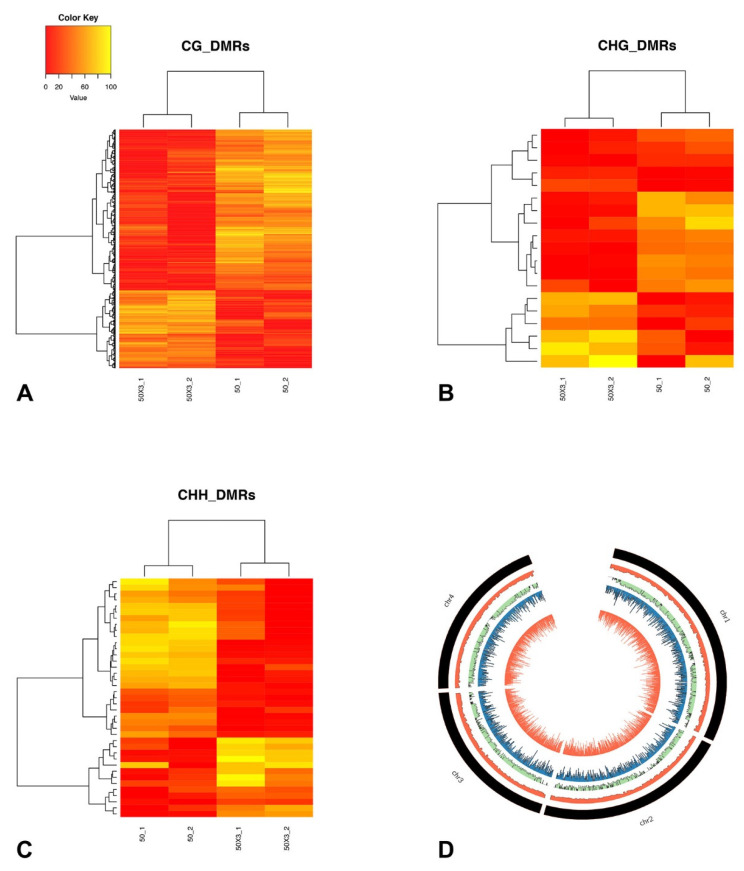
Relative methylation frequencies of differentially methylated regions as identified from the comparison between SC50 and SC50×3 subcultures. Relative methylation frequencies of the differentially methylated positions contained in each differentially methylated region (**A**–**C**) for CG, CHG, and CHH, respectively) were averaged and used in complete linkage clustering analysis of samples derived from SC50 and SC50×3 based on differentially methylated regions. (**D**) Circos plot. From outer inward: number of genes in adjacent genomic chromosome regions of 20 kb. Ratio of the number of *F. graminearum-F. verticillioides* collinear genes and total number of *F. graminearum* genes in each region. DMPs (CG context) and DMRs (CG context).

The estimated relative methylation level of the DMPs belonging to each DMR were hierarchically clustered and, as expected, clustered according to the treatment, as SC50×3 or SC50 (Figure 4 and Supplementary Figure S3). In particular, for all three contexts, the number of DMRs with higher methylation levels in the SC50×3 samples (relative to the SC50 samples) was lower than the number of DMRs that showed a lower level in the SC50×3 samples (Figure 4 and Supplementary Figure S3).

Next, we analysed the distribution of DMPs and DMRs along *F. graminearum* chromosomes. Several studies proposed that *F. graminearum* genome can be partitioned in a core portion enriched for housekeeping genes and a dispensable portion with a high frequency of pathogenesis and virulence-related genes [42]. The dispensable genomic portions can be identified as regions of low gene collinearity (hereafter, referred to as not conserved (NC) regions) between *F. graminearum* and *F. verticillioides* or *F. oxysporum*. The distributions of both DMPs and DMRs along chromosomes were visualized as the total number of these features for each adjacent genomic window of 20 kb. The black histograms of Figure 3 indicate the location of NC regions in four *F. graminearum* chromosomes. For all the analysed contexts, no preferential accumulation of either DMPs or DMRs between NC regions and other chromosome regions were observed (*p* > 0.05 of a non-parametric permutation test).

#### 3.4.2. Differentially Methylated Genes

DMP and DMR distributions were analysed in relation to the coding and regulatory genomic sequences. In particular, we compared the distribution of DMPs and DMRs in transcribed genic regions extended by 0.5 kb at both ends (extended gene bodies, EGBs) (Supplementary Figure S1) and found that DMRs mapped preferentially to EGBs.

**Figure 5:**
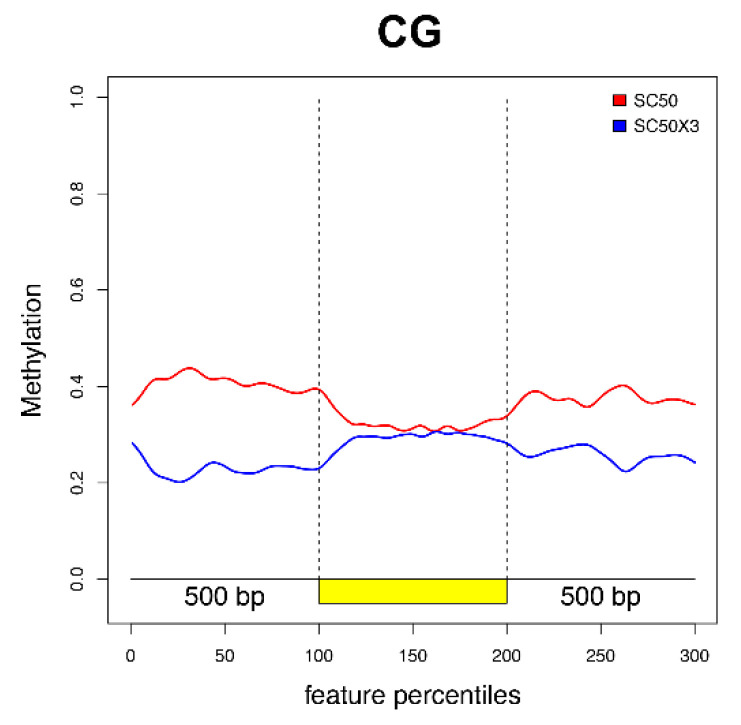
Plotted DMPs along the EGBs (coding region, in yellow, with the regions 500 bp upstream and downstream) for the CG context.

In addition, we plotted the distribution of significant DMPs along the EGBs for CG (Figure 5), CHG, and CHH (Supplementary Figure S4) contexts. The main differences in CG relative methylation levels between SC50 and SC50×3 were observed in the regulative regions and in proximity of TSS and TTS sites. In particular, a decreased, relative, methylation level of the samples grown on plants as compared to artificial substrate was found. Conversely, along the gene body, no changes were highlighted. In the other two contexts, the low number of significant DMPs (Supplementary Table S4) were not able to properly reveal differences along EGB’s genomic region (Supplementary Figure S4).

In particular 930, 13, and 40 EGBs were overlapped at least once by 932 DMRs in the 0.5-kb windows upstream of TSS, within the gene body, or in the 0.5-kb windows downstream of TTS, respectively. The genes belonging to these EGBs were defined as differentially methylated genes (DMGs, Supplementary Table S6).

Analysis of Gene Ontologies’ enrichment demonstrated that DMGs are enriched for GO terms in relation to transcriptional regulation (GO_0006355) and chitin metabolism (GO:0006032). More DMGs than expected by chance were involved in zinc ion (GO:0008270) and DNA (0003677) binding. Next, we assigned DMGs to gene families based on the presence of PFAM domains within the encoded proteins. The PFAM domains are significantly more abundant than expected by chance in the DMGs’ dataset are reported in Table S7 (Fisher exact test *p* < 0.05). Several of these domains have been identified in genes known to be linked directly or indirectly to virulence.

The top five PFAM domains enriched in the DMGs were associated with isoprenes and carbohydrate metabolism (PF08544.13, PF00180.20), endonuclease and exonuclease activity (PF003372), vacuolar 14 fab1-binding (PF12755.7), and ureo-hydrolase (PF00491.21).

## 4. Discussion

Fungal pathogens are the predominant causal agents of plant diseases, causing yield and quality losses [43,44,45]. To successfully infect plants, fungal pathogens use different strategies to exert their virulence during the infection process, such as when adjusting the activity of various molecules, which may be effectors or extracellular factors [46,47], by regulating their transcription levels [20]. Some virulence factors are upregulated to facilitate host colonization and infection, whereas others are downregulated to mitigate host responses [48,49]. Furthermore, the genome plasticity of fungal plant pathogens allows the adaptation of the metabolism and of the reproductive strategies to variable environmental conditions, such as light cycle, temperature, substrate type, and the presence/absence of hosts [50,51]. DNA methylation is a basic modification of genomic DNA in eukaryotes with significant effects on gene expression, genomic imprinting, and transposon silencing involving gene promoter regions, transposable elements, repeat sequences, and transcribed regions of genes [52,53,54,55,56]. In filamentous fungi, transcriptome and methylome studies have shown that DNA methylation is linked to gene expression and to the silencing of transposable elements [52,57]. For these reasons, the present study was based on the DNA methylation approach to reveal hypothetical genes involved in the virulence of *F. graminearum*, which is the most important FHB pathogen of wheat worldwide. In fact, DNA methylation studies can be considered useful tools to identify novel genes associated with the aggressiveness of fungal pathogens subject to environmental modifications [58] and/or stresses, such as adapting to an artificial substrate or to host tissues. Changes of DNA methylation patterns in response to environmental stresses were observed in plants, during their growth and development [53,59,60].

In the present work, a high virulent strain of *F. graminearum* (FG8) was preliminarily stressed when performing subculturing (50 times for 50 weeks) on an artificial substrate (PDA) to verify if the adaptation to a nutrient-rich medium induced an attenuation of virulence. To assess the effect of the *in vitro* subculturing process on fungal virulence, the aggressiveness of three selected subcultures (SC1–SC23–SC50) was examined. Stem base crown rot virulence assays showed a progressive, aggressive decline related to subculturing time toward this bread wheat tissue. A similar result was also observed on bread wheat heads. Furthermore, the mycelium from the subculture developed for 50 weeks on PDA (SC50) was used to inoculate for three consecutive head-to-head passages (SC50×3) bread wheat heads with the objective of restoring the native virulence of the FG8 strain. In fact, SC50×3 exhibited a strong aggressiveness on heads, that is even higher than SC1, likely due to the repeated inoculation of healthy heads in a short time window (6 weeks).

These results are in line with previous studies that showed a virulence decline of different entomopathogenic fungal species or isolates (e.g., *Metarhizium anisopliae*, *Beauveria bassiana*, *Beauveria densa*, *Nomuraea rileyi*, *Paecilomyces farinosus*, *Verticillium lecanii*), caused by artificial *in vitro* subculturing on nutrient-reach media and long-term routine maintenance [61,62,63,64], even if this decline is not always reported, such as in some strains of *Paecilomyces fumosoroseus*, *P. farinosus*, and *B. bassiana* [65,66,67]. As observed in this study, other researchers reported that virulence can be restored when a pathogen passes from an artificial media to a suitable host [68,69,70,71]. In addition to the subculturing process, the simple *in vitro* growth on different artificial media can influence conidial germination, growth, and virulence of fungal pathogens [72,73,74]. The *in vitro* growth on artificial media could also induce genetic modifications, as already demonstrated for a *F. verticillioides* strain subject to a subculturing process, in which the accumulation of about 14 genetic variants was observed [75]. The present work also shows that consecutive *in vitro* subcultures of *F. graminearum* caused a considerable decline of conidiation passing from SC1 to SC50, confirming previous studies on *B. bassiana*, *M. anisopliae*, and *Metarhizium brunneum* strains [76,77]. With the passage on a healthy host, a significant increase of conidia production from SC50 to SC50×3 was observed. Even if some phenotypic changes, such as the reduction in the growth rate, may be typically associated with *in vitro* degeneration [69]. No differences were observed comparing the measures of the diameters of the subcultures, as also found in *B. bassiana* [76].

Finally, a very large spectrum of *F. graminearum* secondary metabolites biosynthesis following prolonged subculturing was investigated. Even if the lower biosynthesis of some secondary metabolites is known to be linked to subculturing processes in different fungal genera, such as *Periconia* sp., *Fusarium* spp., *Galactomyces* sp., and *Phomopsis* sp. [78,79,80], the exact mechanisms that cause this attenuation are still unclear. They may be attributed to the absence of a host stimulus or to gene silencing occurring in axenic cultures [81,82]. However, this phenomenon was not observed in this study.

DNA methylation and transposon activity have already been investigated to be at the base of virulence loss and conidiation ability as a consequence of serial *in vitro* subcultures as well as at the origin of virulence restoration following host re-inoculation [69]. In the present study, we detected a lower level of relative methylation of SC50×3 when compared to SC50 in all contexts, suggesting that this genomic strategy was employed by *F. graminearum* in order to restore an efficient virulence. No significant differences in accumulating methylation changes were observed between genomic compartments. This finding suggests that, to achieve the phenotypic changes described in this study, both genomic regions hosting genes involved in basal metabolism and those regulating virulence are equally important. At a lower scale, the regulatory regions of genes were mainly affected by the previously mentioned methylation changes, especially for the CG context. Moreover, the different number of significant DMPs (Supplementary Table S4) revealed that CG was the methylation context mainly affected by the subculturing process. Differentially methylated region distributions in relation to coding and regulatory genomic sequences identified a total of 1024 genes, which are putatively regulated by DNA methylation.

Some of these genes have already been investigated for their crucial role in *F. graminearum* aggressiveness. Hereafter, some examples of genes that showed different methylation levels between SC50 and SC50×3 with our analyses and that have been previously described in other studies for their role in *F. graminearum* virulence are discussed. The genes FGSG_06675 (FgLeu2A) and FGSG_10671 (FgLeu2B) are known to be involved in the leucine metabolic pathway [83] of *F. graminearum*, and their importance in pathogenicity (in particular of FGSG_06675) and DON production (both FGSG_06675 and FGSG_10671) is well-known [84]. In the present experiment, other genes with different methylation levels during wheat infection are involved in the DON biosynthetic pathway: FGSG_05912 (mevalonate kinase) and FGSG_09764 (5′-phosphomevalonate kinase). These enzymes are responsible for the transformation of mevalonate in 5′-phosphomevalonate and, subsequently, in 5′-pyrphosphomevlonate during the chemical conversion of the acetyl-CoA in farnesyl pyrophosphate (FPP), which is the main substrate for DON biosynthesis [85]. Considering that DON is a well-known virulence factor of *F. graminearum* [86,87], likely FGSG_05912 and FGSG_09764 could be indirectly involved in fungal virulence due to their crucial role in DON production. Another interesting gene differentially methylated between SC50 and 50SCx3 is FGSG_07896. Even if not directly implicated in fungal virulence, this gene encodes for a trichothecene 3-O-acetyltransferase (TRI101) involved in the DON self-protection mechanism of *F. graminearum* [88,89]. In addition, the target of rapamycin (TOR) kinase gene (FGSG_08133) showed different methylation between the two samples. The protein FgTOR encoded by this gene is a key component of the TOR complex. The TOR signaling pathway of *F. graminearum* plays critical roles in regulating vegetative differentiation and virulence [90]. Other differentially methylated genes are FGSG_07067 and FGSG_06944, which encode for two transcription factors. *F. graminearum* mutants with the deletion of these genes showed a significant loss of virulence toward wheat heads [91]. Varied methylation levels of FGSG_07593, encoding a glycoside hydrolase, were observed between SC50×3 and SC50. This gene is usually upregulated at the beginning of the host colonization process [92]. Another gene differentially methylated comparing the two samples is FGSG_11955. The gene was previously identified like the velvet gene (FgVe1 or FgVeA) of *F. graminearum*, a domain conserved in various genera of filamentous fungi. The velvet gene regulates the trichothecene biosynthesis and pathogenicity against wheat heads and affects fungal development [93,94]. In addition to this gene, FGSG_01362 and FGSG_06774 belonged to the velvet gene domain, and, in this study, have shown different methylation levels. FGSG_01973, FGSG_09917, and FGSG_06175 encode for phospholipid hydrolases (phospholipase D, PLD) of *F. graminearum* (FgPLD1, FgPLD2, and FgPLD3). FgPLD1 is involved in the virulence toward flowering wheat heads and the mutant lacking this gene also showed reduced DON production, whereas FgPLD2 and FgPLD3 are not primarily involved in plant infection [95]. The differentially methylated FGSG_05902 gene between SC50×3 and SC50, which is almost identical with FGL15 cloned in previous research, encodes for a lipase known to be an important virulence factor [96]. Again, gene FGSG_04580, encoding a pleiotropic drug resistance class ABC transporter, is known to play a role in *F. graminearum* virulence toward different wheat tissues [97]. Furthermore, the ATP-binding cassette transporter Abc1, encoded by this gene, may be involved in DON release [98]. Several other ABC-G family transporters are highly expressed during host infection, such as FGSG_08309, which showed a different methylation between the two subcultures investigated [97]. The gene FGSG_09329, encoding an ABC-2 family transporter protein, showed a high expression during barley heads and wheat coleoptile colonization [97]. Recently, another ATP-binding cassette transporter (FgArb1) encoded by FGSG_04181, differentially methylated between SC50 and SC50×3, proved to have a function in pathogenesis and DON production [99]. In general, the ABC transporters family has a crucial role in *F. graminearum* pathogenicity [97,100]. Comparing SC50×3 to SC50, FGSG_01964 (GzCHS5), which encodes for a chitin synthase, is indispensable for perithecia formation and pathogenicity as well as for normal sept formation and hyphal growth [101]. Another chitin synthase gene, which is known to be involved both in DON synthesis and pathogenicity [102], is FGSG_06550 that showed different methylation levels between the two explored subcultures, such as FGSG_03538 (transcription factor Tri10) that is essential for DON production, and regulates the expression of the entire Tri-cluster [103,104]. FGSG_00352, differentially methylated between SC50 and SC50×3, is the orthologous protein of Hap2, which is one of the three subunits composing the heme activator protein (HAP), also known as a nuclear factor Y (NF-Y) or CCAAT-binding factor (CBF). *F. graminearum* has eight different genes encoding for CCAAT-binding factors. The deletion of FGSG_00352 did not significantly affect fungal mycelium growth, sexual development, mycotoxin production, and virulence [91], but other CCAAT-binding factors (FGSG_01182 and FGSG_05304) are involved in trichothecene production and virulence [105]. Thus, further studies on all the genes of the CCAAT-binding complex are necessary to reveal the relationship among them during host colonization. Another aggressiveness-associated gene (FGSG_08010), that is, a regulatory virulence [106], and is usually reported to be up-regulated during infection [107], showed a different level of methylation in the present work. A different methylation between the two analyzed subcultures was observed in FGSG_00332, encoding for a beta transducing-like (WD-40 repeat) protein, that has been demonstrated to be essential for pathogenicity in wheat [108], and in FGSG_01665 (FSR1) that regulates *F. graminearum* virulence by acting as a scaffold for a signal transduction pathway [109]. Comparing SC50 to SC50×3, a different methylation level was observed in FGSG_06798. Recently, this gene has been identified to encode for an acetyltransferase (FgHAT2) involved in regulating vegetative growth, conidiation, DNA damage repair, DON production, and virulence in the pathogen [110]. Another highlighted gene previously proven to be involved in pathogenicity was FGSG_00416, belonging to a major facilitator superfamily (MFS) [111]. Furthermore, the deletion of FGSG_03716 (Famfs1), which belongs to the MFS gene family, affected fungal development and virulence [112]. FGSG_03541 (Tri12), with different methylations between the two subcultures, is required for DON production and fungal virulence [113].

In addition, it has been demonstrated that FGSG_03168 has 90% similarity to FST1 of *F. verticillioides* (putative hexose transporter gene), which is functional in pathogenesis during the colonization of living maize kernels [114].

In the present study, we demonstrated that *F. graminearum* exhibits reduced virulence on bread wheat stem bases and heads after a prolonged subculturing process. However, the virulence on head tissue of bread wheat can be restored with the in planta transfer. Additionally, an innovative approach, based on the relative methylation level analysis, was used to explore novel putative virulence genes, comparing the pathogen after three generations of mycelium growth on bread wheat heads to the same fungus after approximately one-year of an *in vitro* subculturing process. Some of the genes that showed different methylation levels have been previously studied and were revealed to be related to *Fusarium* aggressiveness toward hosts. This suggests that the approach of the present study could be a promising tool in the study of *F. graminearum* genes associated with virulence on bread wheat tissues. In the future, it will be interesting to verify the function and possible involvement in virulence of all the other genes that have shown different methylation levels in this study (listed in Supplementary Table S7) but for which evidence about their implication in *F. graminearum* aggressiveness are not currently available. Finally, the present approach may be an important tool to use for other fungal pathogens to explore the pool of genes that could be involved in their virulence toward host species.

## Supplementary Materials

The following materials are available online at https://www.mdpi.com/article/10.3390/cells10051192/s1. Table S1: *In vitro* biosynthesis of secondary metabolites (μg kg^−1^) produced by subcultures of *F. graminearum* strain FG8 (SC1–SC23–SC50–SC50×3) as detected by LC-MS/MS analysis. Per each mycotoxin, the standard error (SE) is reported. Statistical differences were detected (*p* > 0.05) by one-way ANOVA. Table S2: Experimental design. Table S3: Sequencing data summary. Table S4: Methylation sequencing statistics. Uniquely mapped loci (MCSeEd loci) were normalized, filtered, and then analysed with the methyl kit to infer the number of differentially methylated positions (DMPs). Figure S1: Abundance of MCSeEd loci, DMPs, and DMRs in genomic regions (CDS, Introns, Regulatory Intergenic). Figure S2: Principal component analysis and clustering for the relative methylation levels at the differentially methylated positions obtained across all of the replicates in each sequence context: CG, CHG, and CHH. Number in brackets indicate the fraction of overall variance explained by the respective component (Dim1, Dim2). Table S5: List of significant DMRs identified in each context. Figure S3: Principal component analysis and boxplot for the relative methylation levels at the differentially methylated positions obtained across all of the replicates in each sequence context: CG, CHG, and CHH. Numbers in brackets indicate the fraction of overall variance explained by the respective component (Dim1, Dim2). Figure S4: Plotted DMPs along the EGBs (coding region, in yellow, with the regions 500 bp upstream and downstream) for CHG and CHH context. Table S6: Differentially methylated genes (DMGs) identified by intersecting DMRs. Table S7: Enrichment tests of PFAM domains within DMGs and *F. graminearum* loci. First column reports the PFAM domain, the second column reports the odds ratio, and the third column shows the *p*-value calculated for the Fisher exact test for an odd ratio equal to 1 (alternative hypothesis odds ratio >1).

## References

[1] O’Donnell K., Rooney A.P., Proctor R.H., Brown D.W., McComick S.P., Ward T.J., Frandsen R.J.N., Lysoe E., Rehner S.A., Aoki T. (2013). Phylogenetic analyses of RPB1 and RPB2 support a middle Cretaceous origin for a clade comprising all agriculturally and medically important fusaria. Fungal Genet. Biol..

[2] Goswami R.S., Kistler H.C. (2004). Heading for disaster: *Fusarium graminearum* on cereal crops. Mol. Plant Pathol..

[3] Kazan K., Gardiner D.M., Manners J.M. (2012). On the trace of a cereal killer: Recent advances in *Fusarium graminearum* pathogenomics and host resistance. Mol. Plant Pathol..

[4] Mielniczuk E., Skwarylo-Bednarz B. (2020). Fusarium head blight, mycotoxins and strategies for their reduction. Agronomy.

[5] Geraldo M.R.F., Tessmann D.J., Kemmelmeier C. (2006). Production of mycotoxins by *Fusarium graminearum* isolated from small cereal (wheat, triticale and barley) affected with scab disease on Southern Brazil. Braz. J. Microbiol..

[6] Ward T.J., Bielawski J.P., Kistler H.C., Sullivan E., O’Donnel K. (2002). Ancestral polymorphism and adaptive evolution in the trichothecene mycotoxin gene cluster of phytopathogenic Fusarium. Proc. Natl. Acad. Sci. USA.

[7] Feil R., Fraga M.F. (2012). Epigenetics and the environment: Emerging patterns and implications. Nat. Rev. Genet..

[8] Causevic A., Delaunay A., Ounnar S., Righezza M., Delmotte F., Brignolas F., Hagege D., Maury S. (2005). DNA methylating and demethylating treatments modify phenotype and cell wall differentiation state in sugar beet cell lines. Plant Physiol. Biochem..

[9] Cao D., Gao X., Liu J., Wang X., Geng S., Yang C., Liu B., Shi D. (2012). Root-specific DNA methylation in *Chloris virgata*, a natural alkaline-resistant halophyte, in response to salt and alkaline stresses. Plant Mol. Biol. Rep..

[10] Angers B., Castonguay E., Massicotte R. (2010). Environmentally induced phenotypes and DNA methylation: How to deal with unpredictable conditions until the next generation and after. Mol. Ecol..

[11] Lu Y., Rong T., Cao M. (2008). Analysis of DNA methylation in different maize tissues. J. Genet. Genom..

[12] Lizal P., Relichova J. (2001). The effect of day length, vernalization and DNA demethylation on the flowering time in *Arabidopsis thaliana*. Physiol. Plant..

[13] Seidl M.F. (2017). Adenine N6-methylation in diverse fungi. Nat. Genet..

[14] Mondo S.J., Dannebaum R.O., Kuo R.C., Louie K.B., Bewick A.J., Labutti K., Haridas S., Keu A., Salamov A., Ahrendt S.R. (2017). Widespread adenine N6- methylation of active genes in fungi. Nat. Genet..

[15] Lang Z., Wang Y., Tang K., Tang D., Datsenka T., Cheng J., Zhang J., Handa A.K., Zhu J.K. (2017). Critical roles of DNA demethylation in the activation of ripening-induced genes and inhibition of ripening-repressed genes in tomato fruit. Proc. Natl. Acad. Sci. USA.

[16] Zhang X., Yazaki J., Sundaresan A., Cokus S., Chan S.W.L., Chen H., Henderson I.R., Shinn P., Pellegrini M., Jacobsen S.E. (2006). Genome-wide high-resolution mapping and functional analysis of DNA methylation in Arabidopsis. Cells.

[17] Goll M.G., Bestor T.H. (2005). Eukaryotic cytosine methyltransferases. Annu. Rev. Biochem..

[18] Paszkowski J., Whitham S.A. (2001). Gene silencing and DNA methylation processes. Curr. Opin. Plant Biol..

[19] Suzuki M.M., Bird A. (2008). DNA methylation landscapes: Provocative insights from epigenomics. Nat. Rev. Genet..

[20] He C., Zhang Z., Li B., Tian S. (2020). The pattern and function of DNA methylation in fungal plant pathogens. Microorganisms.

[21] Freitag M., Williams R.L., Kothe G.O., Selker E.U. (2002). A cytosine methyltransferase homologue is essential for repeat-induced point mutation in *Neurospora crassa*. Proc. Natl. Acad. Sci. USA.

[22] Malagnac F., Wendel B., Goyon C., Faugeron G., Zickler D., Rossignol J.L., Noyer-Weidner M., Vollmayr P., Trautner T.A., Walter J. (1997). A gene essential for de novo methylation and development in *Acobolus* reveals a novel type of eukaryotic DNA methyltransferase structure. Cell.

[23] Bewick A.J., Hofmeister B.T., Powers R.A., Mondo S.J., Grigoriev I.V., James T.Y., Stajich J.E., Schmitz R.J. (2019). Diversity of cytosine methylation across the fungal tree life. Nat. Ecol. Evol..

[24] Freitag M., Hickey P.C., Khlafallah T.K., Read N.D., Selker E.U. (2004). HP1 is essential for DNA methylation in *Neurospora*. Mol. Cell.

[25] Tamaru H., Selker E.U. (2003). Synthesis of signals for de novo DNA methylation I *Neurospora crassa*. Mol. Cell Biol..

[26] Kouzminova E., Selker E.U. (2001). dim-2 encode a DNA methyltransferase responsible for all known cytosine methylation in *Neurospora*. EMBO J..

[27] Ma L.J., van der Does H.C., Borkovich K.A., Coleman J.J., Daboussi M.J., Di Pietro A., Dufresne M., Freitag M., Grabherr M., Henrissat B. (2010). Comparative genomics reveals mobile pathogenicity chromosomes in *Fusarium*. Nature.

[28] Pomraning K.R., Connolly L.R., Whalen J.P., Smith K.M., Freitag M., Brown D., Proctor R.H. (2013). Repeat-induced point mutation, DNA methylation and heterochromatin in *Gibberella zeae* (Anamorph: *Fusarium graminearum*. Fusarium Genomics and Molecular Cellular Biology.

[29] Gijezen M., Ishmael C., Shrestha D. (2014). Epigenetic control of effectors in plant pathogens. Front. Plant Sci..

[30] Kim D.H. (1997). Induced change in DNA methylation of *Fusarium oxysporum* f. sp. niveum due to successive transfer. J. Biochem. Mol. Biol..

[31] Covarelli L., Beccari G., Prodi A., Generotti S., Etruschi F., Juan C., Ferrer E., Mañes J. (2015). *Fusarium* species, chemotype characterization and trichothecene contamination of durum and soft wheat in an area of central Italy. J. Sci. Food Agric..

[32] Simpson D.R., Rezanoor H.N., Parry D.W., Nicholson P. (2000). Evidence for differential host preference in *Microdochium nivale* var. *majus*, *M. nivale* var. *nivale*. Plant Pathol..

[33] Beccari G., Covarelli L., Nicholson P. (2011). Infection processes and soft wheat response to root rot and crown rot caused by *Fusarium culmorum*. Plant Pathol..

[34] Covarelli L., Beccari G., Steed A., Nicholson P. (2012). Colonization of soft wheat following infection of the stem base by *Fusarium culmorum* and translocation of deoxynivalenol to the head. Plant Pathol..

[35] Brunner K., Lichtenauer A.M., Kratochwill K., Delic M., Mach R.L. (2007). Xyr1 regulates xylanase but not cellulase formation in the head blight fungus *Fusarium graminearum*. Curr. Genet..

[36] Sulyok M., Stadler D., Steiner D., Krska R. (2020). Validation of an LC-MS/MS-based diluted-and-shoot approach for the quantification of >500 mycotoxins and other secondary metabolites in food crops: Challenges and solutions. Anal. Bioanal. Chem..

[37] Marconi G., Capomaccio S., Comino C., Acquadro A., Portis E., Porceddu A., Albertini E. (2019). Methylation content sensitive enzyme ddRAD (MCSeEd): A reference-free, whole genome profiling system to address cytosine/adenine methylation changes. Sci. Rep..

[38] Di Marsico M., Cerruti E., Comino C., Porceddu A., Acquadro A., Capomaccio S., Marconi G., Albertini E., Spillane C., McKeown P. (2020). MCSeEd (Methylation Context Sensitive Enzyme ddRAD): A new method to analyze DNA methylation. Plant Epigenetics and Epigenomics. Methods in Molecular Biology.

[39] Akalin A., Kormaksson M., Li S., Garrett-Bakelman F.E., Melnick A., Mason C.E. (2012). MethylKit: A comprehensive R package for the analysis of genome-wide DNA methylation profiles. Genome Biol..

[40] Onofri A., Pannacci E. (2014). Spreadsheet tools for biometry classes in crop science programmes. CBCS.

[41] Cuomo C.A., Guldener U., Xu J.R., Trail F., Turgeon B.G., Di Pietro A., Walton J.D., Baker S.E., Rep M., Adam G. (2007). The *Fusarium graminearum* genome reveals a link between localized polymorphism and pathogen specialization. Science.

[42] Zhao C., Waalwijk C., de Wit P.J.G.M., Tang D., van der Lee T. (2014). RNA-Seq analysis reveals new gene models and alternative splicing in the fungal pathogen *Fusarium graminearum*. BMC Genom..

[43] Li B.Q., Zong Y.Y., Du Z.L., Shang Y.J., Chen Y., Zhang Z.Q., Qin G.Z., Zhao W.M., Tian S.P. (2015). Genomic characterization reveals insights into patulin biosynthesis and pathogenicity in *Penicillium* species. Mol. Plant Microbe Interact..

[44] Malcom G.M., Kuldau G.A., Gugino B.K., Jimenez-Gasco M.D.M. (2013). Hidden host plant associations of soilborne fungal pathogens: An ecological perspective. Phytopathology.

[45] Dean R., Kan J.A.V., Pretorius Z.A., Hammond-Kosack K.E., Pietro A.D., Spanu P.D., Rudd J.J., Dickman M., Kahmann R., Ellis J. (2012). The top 10 fungal pathogens in molecular plant pathology. Mol. Plant Pathol..

[46] Presti L.L., Lanver D., Schweizer G., Tanaka S., Liang L., Tollot M., Zuccaro A., Reissmann S., Kahmann R. (2015). Fungal effectors and plant susceptibility. Annu. Rev. Plant Biol..

[47] Presti L.L., Kahmann R. (2017). How filamentous plant pathogen effectors are translocated to host cells. Curr. Opin. Plant Biol..

[48] Oliveira-Garcia E., Valent B. (2015). How eukaryotic filamentous pathogens evade plant recognition. Curr. Opin. Microbiol..

[49] Shalaby S., Horwitz B.A. (2015). Plant phenolic compounds and oxidative stress: Integrated signals in fungal-plant interactions. Curr. Genet..

[50] Reverberi M., Punelli M., Scala V., Scarpari M., Uva P., Mentzen W.I., Dolezal A.L., Woloshuk C., Pinzari F., Fabbri A.A. (2013). Genotypic and phenotypic versatility of *Aspergillus flavus* during maize exploitation. PLoS ONE.

[51] Slepecky R.A., Starmer W.T. (2009). Phenotypic plasticity in fungi: A review with observations on *Aureobasidium pullulans*. Mycologia.

[52] Bartels A., Han Q., Nair P., Stacey L., Gaynier H., Mosley M., Huang Q.Q., Pearson J.K., Hsieh T.F., An Y.C. (2018). Dynamic DNA methylation in plant growth and development. Int. J. Mol. Sci..

[53] Zhong S., Fei Z., Chen Y.R., Zheng Y., Huang M., Vrebalov J., McQuinn R., Gapper N., Liu B., Xiang J. (2013). Single-base resolution methylomes of tomato fruit development reveal epigenome modifications associated with ripening. Nat. Biotechnol..

[54] Moore L.D., Le T., Fan G.P. (2013). DNA methylation and its basic function. Neuropsychopharmacology.

[55] Su Z.X., Han L., Zhao Z.M. (2011). Conservation and divergence of DNA methylation in eukaryotes: New insights from single base resolution DNA methylomes. Epigenetics.

[56] Bird A. (2002). DNA methylation patterns and epigenetic memory. Genes Dev..

[57] Howlett B.J., Lowe R.G.T., Marcroft S.J., van de Wouw A.P. (2015). Evolution of virulence in fungal plant pathogens: Exploiting fungal genomics to control plant disease. Mycologia.

[58] Jeon J., Choi J., Lee G.W., Park S.Y., Huh A., Dean R., Lee Y.H. (2015). Genome-wide profiling of DNA methylation provides insights into epigenetic regulation of fungal development in a plant pathogenic fungus, *Magnaporthe oryzae*. Sci. Rep..

[59] An Y.C., Goettel W., Han Q., Bartels A., Liu Z., Xiao W. (2017). Dynamic changes of genome-wide DNA methylation during soybean seed development. Sci. Rep..

[60] Cokus S.J., Feng S.H., Zhang X.Y., Chen Z.G., Merriman B., Haudenschild C.D., Pradhan S., Nelson S.F., Pellegrini M., Jacobsen S.E. (2008). Shotgun bisulphite sequencing of the *Arabidopsis* genome reveals DNA methylation patterning. Nature.

[61] Nahar P.B., Kulkarani S.A., Kulye M.S., Chavan S.B., Kulkarani G., Rajendran A., Yadav P.D., Shouche Y., Deshpande M.V. (2008). Effect of repeated *in vitro* subculturing on the virulence of *Metarhizium anisopliae* against *Helicoverpa armigera* (Lepidoptera: Noctuidae). Biocontrol Sci. Technol..

[62] Shah F.A., Allen N., Wright C.J., Butt T.M. (2007). Repeated *in vitro* subculturing alters spore surface properties and virulence of *Metarhizium anisopliae*. FEMS Microbiol. Lett..

[63] Morrow B.J., Boucias D.G., Heath M.A. (1989). Loss of virulence in an isolate of an entomopathogenic fungus, *Nomuraea rileyi*, after serial *in vitro* passages. J. Econ. Entomol..

[64] Ignoffo C.M., McIntosh A.H., Garcia C., Kroha M., Johnson J.M. (1982). Effects of successive *in vitro* and *in vivo* passages on the virulence of the entomopathogenic fungus, *Nomuraea rileyi*. Entomophaga.

[65] Vandenberg J.D., Cantone F.A. (2004). Effect of serial transfer of three strains of *Paecilomyces fumosoroseus* on growth *in vitro*, virulence and host specificity. J. Invertebr. Pathol..

[66] Brownbridge M., Costa S., Jaronski S.T. (2001). Effects of *in vitro* passage of *Beauveria bassiana* on virulence to *Bemisia argentifolii*. J. Invertebr. Pathol..

[67] Hayden T.P., Bidochka M.J., Khachatourians G.C. (1992). Entomopathogenicity of several fungi toward the English grain aphid (Homoptera: Aphididae) and enhancement of virulence with host passage of *Paecilomyces farinosus*. J. Econ. Entomol..

[68] Hussain A., Tian M.Y., He Y.R., Lei Y.Y. (2010). Differential fluctuation in virulence and VOC profiles among different cultures of entomopathogenic fungi. J. Invertebr. Pathol..

[69] Butt T.M., Wang C.S., Shah F.A., Hall R., Eilenberg J., Hokkanen H.M.T. (2006). Degeneration of entomogenous fungi. An Ecological and Societal Approach to Biological Control.

[70] Shah F.A., Wang C.S., Butt T.M. (2005). Nutrition influences growth and virulence of the insect-pathogenic fungus *Metarhizium anisopliae*. FEMS Microbiol. Lett..

[71] Butt T.M., Goettel M., Navon A., Ascher K.R.S. (2000). Bioassays of entomopathogenic fungi. Bioassays of Entomopathogenic Microbes and Nematodes.

[72] Hussain A., Tian M.Y., He Y.R., Ruan L., Ahmed S. (2010). *In vitro* and in vivo culturing impacts on the virulence of characteristics of serially passed entomopathogenic fungi. J. Food Agric. Environ..

[73] Hutwimmer S., Wagner S., Affenzeller M., Burgstaller W., Strasser H. (2008). Algorithmbased design of synthetic growth media stimulating virulence properties of *Metarhizium anisopliae* conidia. J. Appl. Microbiol..

[74] Rangel D., Braga G., Flint S.D. (2004). Variations in UV-B tolerance and germination speed of *Metarhizium anisopliae* conidia produced on insects and artificial substrates. J. Invertebr. Pathol..

[75] Scala V., Grottoli A., Cigliano R.A., Anzar I., Beccacioli M., Fanelli C., Dall’Asta C., Battilani P., Reverberi M., Sanseverino W. (2017). Carefully with that axe, gene, genome perturbation after a PEG-mediated protoplast transformation in *Fusarium verticillioides*. Toxins.

[76] Safavi S.A. (2012). Attenuation of the entomopathogenic fungus *Beauveria bassiana* following serial *in vitro* transfers. Biologia.

[77] Ansari M.A., Butt T.M. (2011). Effects of successive subculturing on stability, virulence, conidial yield, germination and shelf-life of entomopathogenic fungi. J. Appl. Microbiol..

[78] Kusari S., Zuelke S., Spiteller M. (2011). Effect of artificial reconstitution of the interaction between the plant *Camptotheca acuminata* and the fungal endophyte *Fusarium solani* on camptothecin biosynthesis. J. Nat. Prod..

[79] Gurudatt P.S., Priti V., Sweta S., Ramesha B.T., Ravikanth G., Vasudeva R., Amna T., Deepika S., Ganeshaiah K.N., Shaanker U.R. (2010). Attenuation of camptothecin production and negative relation between hyphal biomass and camptothecin content in endophytic fungal strains isolated from *Nothapodytes nimmoniana* Grahm (Icacinaceae). Curr. Sci..

[80] Li J., Sidhu R.S., Ford E.J., Long D.M., Hess W.M., Strobel G.A. (1998). The induction of taxol production in the endophytic fungus-*Periconia* sp. from *Torreya grandifolia*. J. Ind. Microbiol. Biotechnol..

[81] Priti V., Ramesha B.T., Shweta S., Ravikanth G., Ganeshaiah K.N., Suryanarayanan T.S., Shaanker U.R. (2009). How promising are endophytic fungi as alternative sources of plant secondary metabolites. Curr. Sci..

[82] Sachin N., Manjunatha B.L., Kumara M.P., Ravikanth G., Shweta S., Suryanarayanan T.S., Ganeshaiah K.N., Shaanker U.R. (2013). Do endophytic fungi possess pathway genes for plant secondary metabolites?. Curr. Sci..

[83] Subramaniam R., Narayanan S., Walkowiak S., Wang L., Joshi M., Rocheleau H., Ouellet T., Harris L.J. (2015). Leucine metabolism regulates TRI6 expression and affects deoxynivalenol production and virulence in *Fusarium graminearum*. Mol. Microbiol..

[84] Liu X., Han Q., Wang J., Wang X., Xu J., Shi J. (2016). Two FgLEU2 genes with different roles in leucine biosynthesis and infection-related morphogenesis in *Fusarium graminearum*. PLoS ONE.

[85] Zheng X., Zhang X., Zhao L., Apaliya M.T., Yang Q., Sun W., Zhang X., Zhang H. (2017). Screening of deoxynivalenol producing strains and elucidation of possible toxigenic molecular mechanism. Toxins.

[86] Bai G.H., Desjardins A.E., Plattner R.D. (2002). Deoxynivalenol-nonproducing *Fusarium graminearum* causes initial infection, but does not cause disease spread in wheat spikes. Mycopathologia.

[87] Eudes F., Comeau A., Rioux S., Collin J. (2001). Impact of trichothecenes on Fusarium head blight (*Fusarium graminearum*) development in jspring wheat (*Triticum aestivum*). Can. J. Plant Pathol..

[88] Kimura M., Kaneko I., Komiyama M., Takatsuki A., Koshino H., Yoneyama K., Yamaguchi I. (1998). Trichothecene 3-O-acetyltransferase protects both the producing organism and transformed yeast from related mycotoxins. Cloning and characterization of Tri101. J. Biol. Chem..

[89] McCormick S.P., Alexander N.J., Trapp S.E., Hohn T.M. (1999). Disruption of TRI101, the gene encoding trichothecene 3-O-acetyltransferase, from *Fusarium sporotrichioides*. Appl. Environ. Microbiol..

[90] Yu F., Gu Q., Yun Y., Yin Y., Xu J.R., Shim W.B., Ma Z. (2014). The TOR signaling pathway regulates vegetative development and virulence in *Fusarium graminearum*. New Phytol..

[91] Son H., Seo Y.S., Min K., Park A.R., Lee J., Jin J.M., Lin Y., Cao P., Hong S.Y., Kim E.K. (2011). A phenome-based functional analysis of transcription factors in the cereal head blight fungus, *Fusarium graminearum*. PLoS Pathog..

[92] Puri K.D., Yan C., Leng Y., Zhong S. (2016). RNA-Seq revealed differences in transcriptomes between 3ADON and 15ADON populations of *Fusarium graminearum in vitro* and *in planta*. PLoS ONE.

[93] Merhej J., Urban M., Dufresne M., Hammond-Kosack K.E., Richard-Forget F., Barreau C. (2011). The velvet gene, FgVe1, affects fungal development and positively regulates trichothecene biosynthesis and pathogenicity in *Fusarium graminearum*. Mol. Plant Pathol..

[94] Jiang J., Liu X., Yin Y., Ma Z. (2011). Involvement of a velvet protein FgVeA in the regulation of asexual development, lipid and secondary metabolisms and virulence in *Fusarium graminearum*. PLoS ONE.

[95] Ding M., Zhu Q., Liang Y., Li J., Fan X., Yu X., He F., Xu H., Ling Y., Yu J. (2017). Differential roles of three FgPLD genes in regulating development and pathogenicity in *Fusarium graminearum*. Fungal Genet. Biol..

[96] Niu X.W., Zheng Z.Y., Feng Y.G., Guo W.Z., Wang X.Y. (2013). The *Fusarium graminearum* virulence factor FGL targets an FKBP12 immunophilin of wheat. Gene.

[97] Gardiner D.M., Stephens A.E., Munn A.L., Manners J.M. (2013). An ABC pleiotropic drug resistance transporter of *Fusarium graminearum* with a role in crown and root diseases of wheat. FEMS Microbiol. Lett..

[98] O’Mara S.P., Broz K., Boenisch M., Zhong Z., Dong Y., Kistler H.C. (2020). The *Fusarium graminearum* t-SNARE Sso2 is involved in growth, defense, and DON accumulation and virulence. Mol. Plant-Microbe Int..

[99] Yin Y., Wang Z., Cheng D., Chen X., Chen Y., Ma Z. (2018). The ATP-binding protein FgArb1 is essential for penetration, infectious and normal growth of *Fusarium graminearum*. New Phytol..

[100] Ammar G.A., Tryono R., Doll K., Karlovsky P., Deising H.B., Wirsel S.G.R. (2013). Identification of ABC transporter genes of *Fusarium graminearum* with roles in azole tolerance and/or virulence. PLoS ONE.

[101] Kim J.E., Lee H.J., Lee J., Kim K.W., Yun S.H., Shim W.B., Lee Y.W. (2009). *Gibberella zeae* chitin synthase genes, GzCHS5 and GzCHS7, are required for hyphal growth, perithecia formation, and pathogenicity. Curr. Genet..

[102] Zhang Y.Z., Chen Q., Liu C.H., Liu Y.B., Yi P., Niu K.X., Wang Y.Q., Wang A.Q., Yu H.Y., Pu Z.E. (2016). Chitin synthase gene FgCHS8 affects virulence and fungal cell wall sensitivity to environmental stress in *Fusarium graminearum*. Fungal Biol..

[103] Seong K.Y., Pasquali M., Zhou X., Song J., Hilburn K., McCormick S., Dong Y., Xu J.R., Kistler H.C. (2009). Global gene regulation by *Fusarium* transcription factors Tri6 and Tri10 reveals adaptations for toxin biosynthesis. Mol. Microbiol..

[104] Guo L., Ji M., Ye K. (2020). Dynamic network inference and association computation discover gene modules regulating virulence, mycotoxin and sexual reproduction in *Fusarium graminearum*. BMC Genom..

[105] Kim J.E., Nam H., Park J., Choi G.J., Lee Y.W., Son H. (2020). Characterization of the CCAAT-binding transcription factor complex in the plant pathogenic fungus *Fusarium graminearum*. Sci. Rep..

[106] Talas F., Kalih R., Miedaner T., McDonald B.A. (2016). Genome-wide association study identifies novel candidate genes for aggressiveness, deoxynivalenol production, and azole sensitivity in natural field populations of *Fusarium graminearum*. Mol. Plant-Microbe Int..

[107] Lawler K., Hammond-Kosack K., Brazma A., Coulson R.M.R. (2013). Genomic clustering and co-regulation of transcriptional networks in the pathogenic fungus *Fusarium graminearum*. BMC Syst. Biol..

[108] Ding S., Mehrabi R., Koten C., Kang Z., Wei Y., Seong K., Kistler H.C., Xu J.R. (2009). Transduction beta-like gene FTL1 is essential for pathogenesis in *Fusarium graminearum*. Eukaryot. Cell.

[109] Shin W.B., Sagaram U.S., Choi Y.E., So J., Wilkinson H.H., Lee Y.W. (2006). FSR1 is essential for fungal virulence and female fertility in *Fusarium verticillioides* and in *Fusarium graminearum*. Mol. Plant Microbe Int..

[110] Lu W.Y., Yang N., Xu Z., Dai H., Tang S., Wang Z.Y. (2020). FgHAT2 is involved in regulating vegetative growth, conidiation, DNA damage repair, DON production and virulence in *Fusarium graminearum*. J. Integr. Agric..

[111] Dufresne M., van der Lee T., M’Barek S.B., Xu X., Zhang X., Liu T., Waalwijk C., Zhang W., Kema G.H.J., Daboussi M.J. (2008). Transposon-tagging identifies novel pathogenicity genes in *Fusarium graminearum*. Fungal Genet. Biol..

[112] Ren W., Zhao H., Shao W., Ma W., Wang J., Zhou M., Chen C. (2016). Identification of a novel phenamacril-resistance-related gene by the cDNA-RAPD method in *Fusarium asiaticum*. Pest Manag. Sci..

[113] Menke J., Dong Y., Kistler H.C. (2012). *Fusarium graminearum* Tri12p influences virulence to wheat and trichothecene accumulation. Mol. Plant Microbe Int..

[114] Kim H., Woloshuk C.P. (2010). Functional characterization of fst1 in *Fusarium verticillioides* during colonization of maize kernels. Mol. Plant Microbe Int..

