# Supplementary figures and images for: Identification of Putative Virulence Genes by DNA Methylation Studies in the Cereal Pathogen Fusarium graminearum
Source: Cells. 2021 May 13;10(5):1192. doi: 10.3390/cells10051192 (PMC8152758; doi:10.3390/cells10051192)

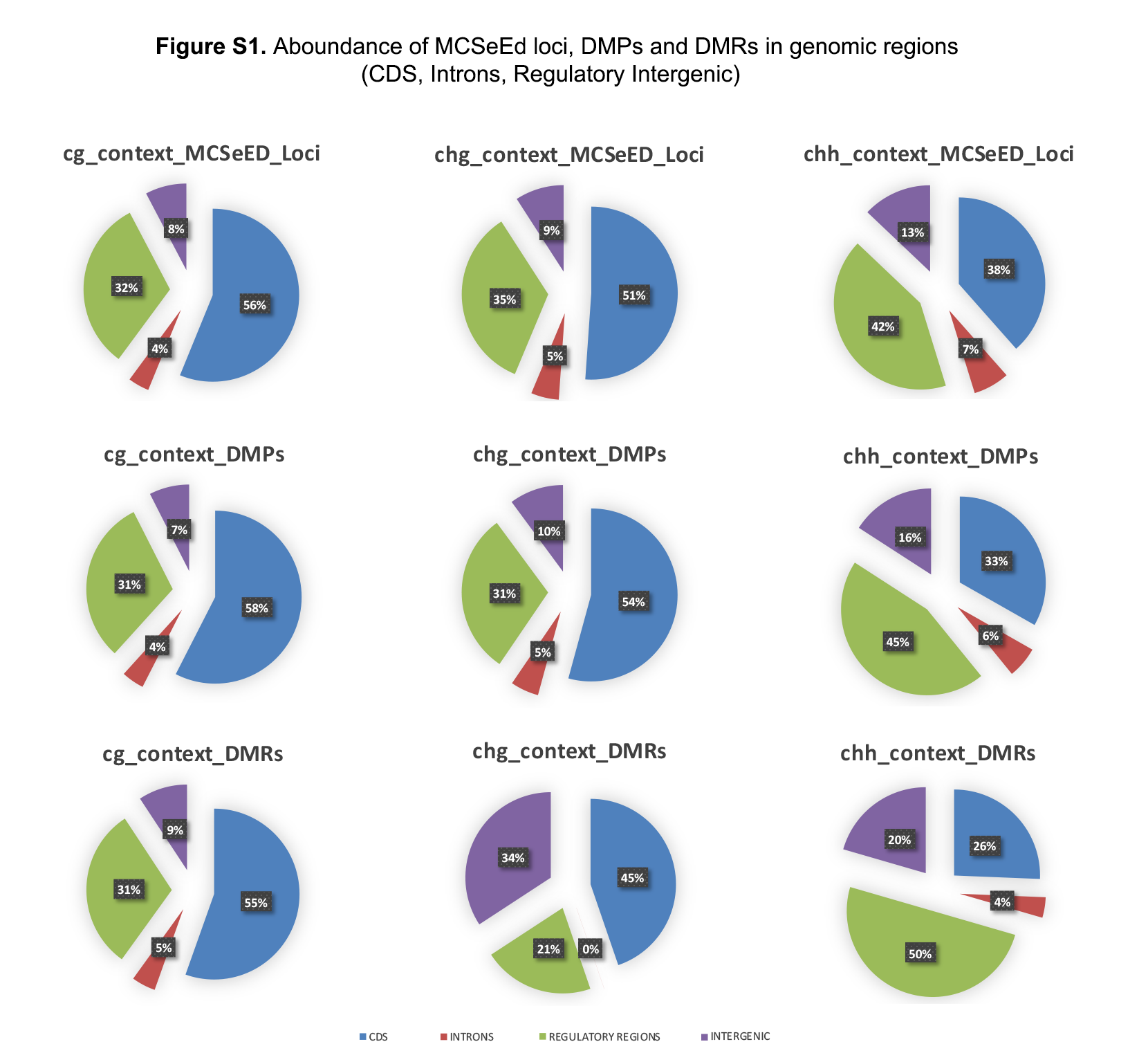

Supplement: Supplementary file 1 [file cells-10-01192-s001.zip › Supplementary files/Figure S1.tif]

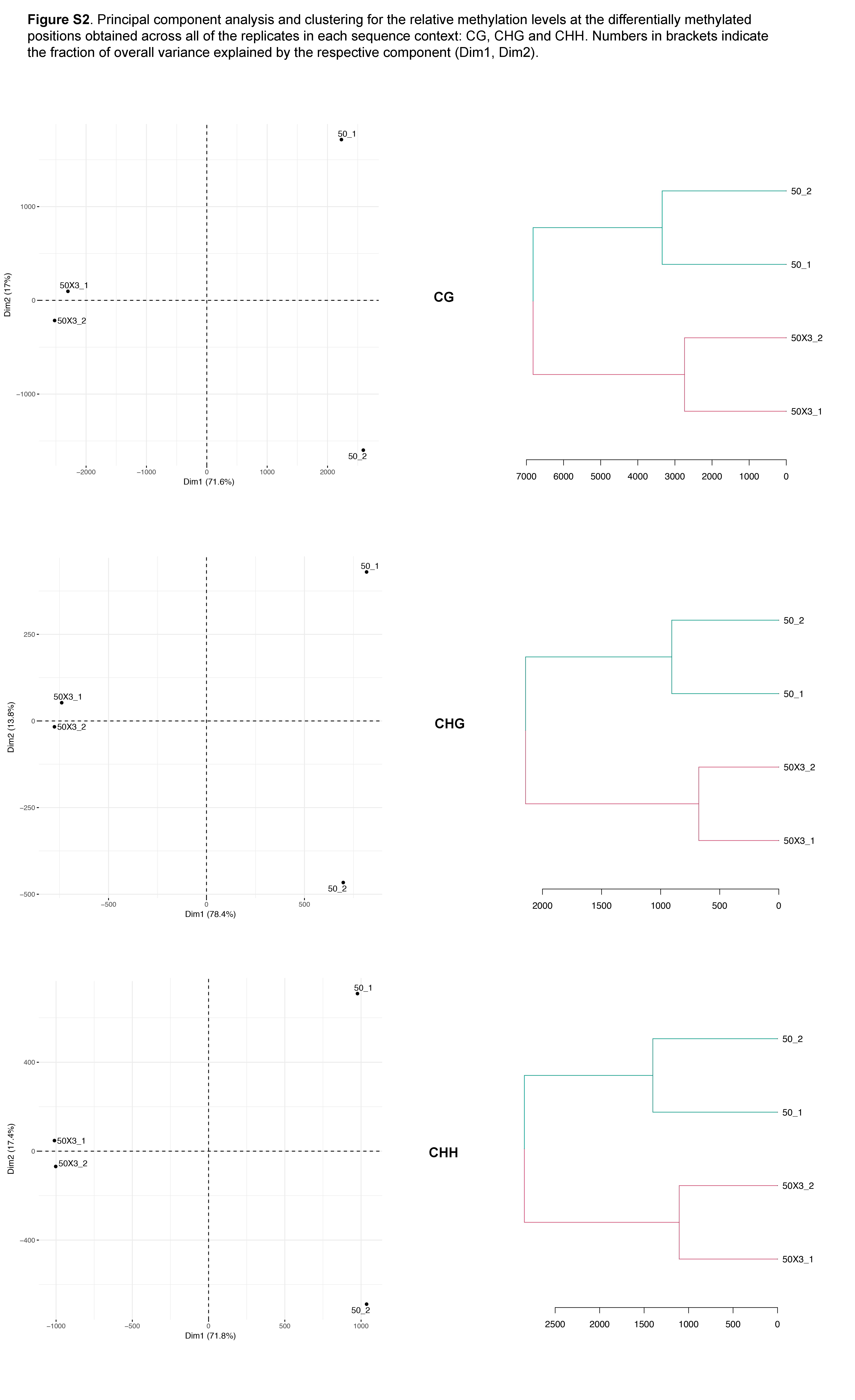

Supplement: Supplementary file 1 [file cells-10-01192-s001.zip › Supplementary files/Figure S2.tif]

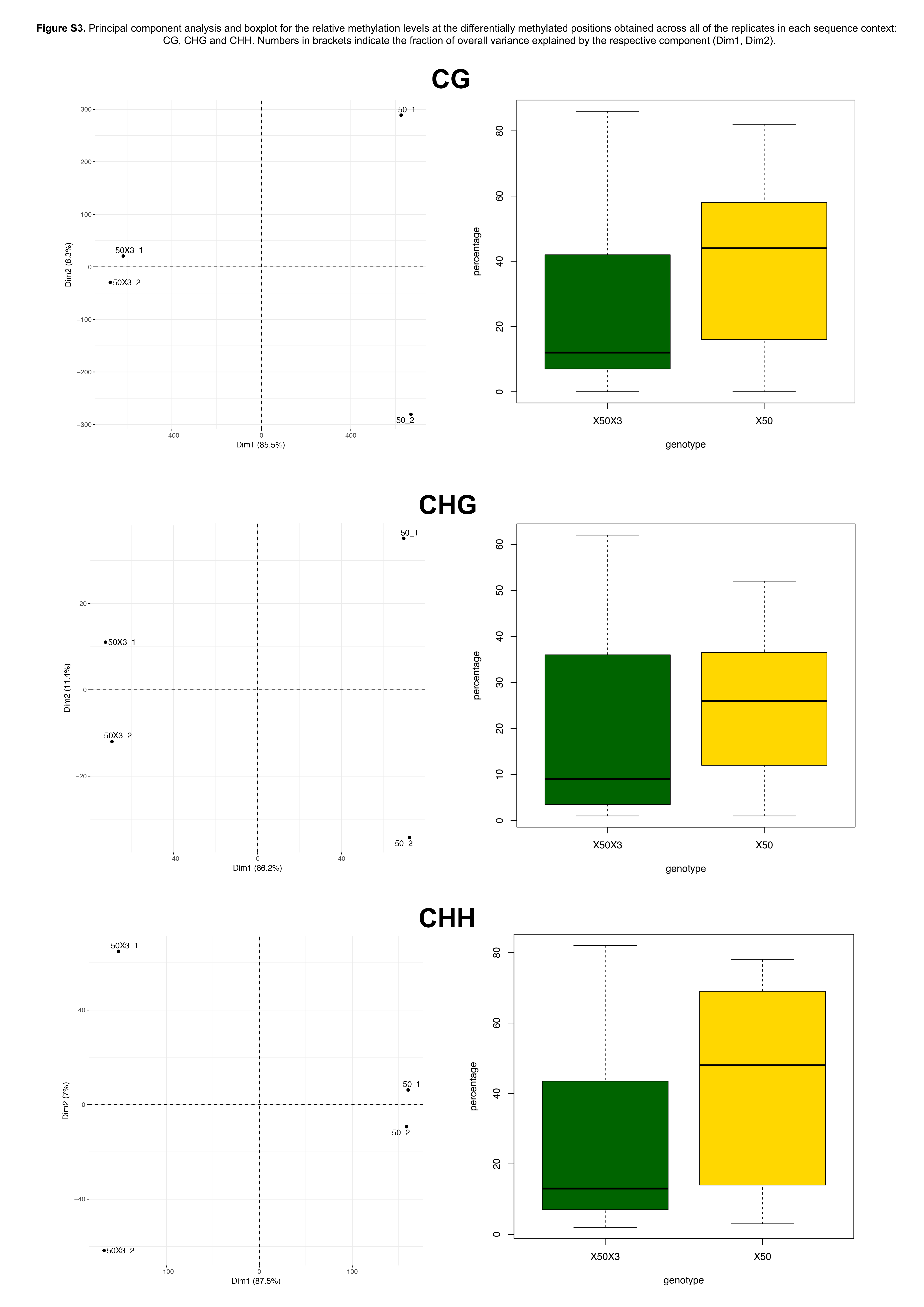

Supplement: Supplementary file 1 [file cells-10-01192-s001.zip › Supplementary files/Figure S3.tif]

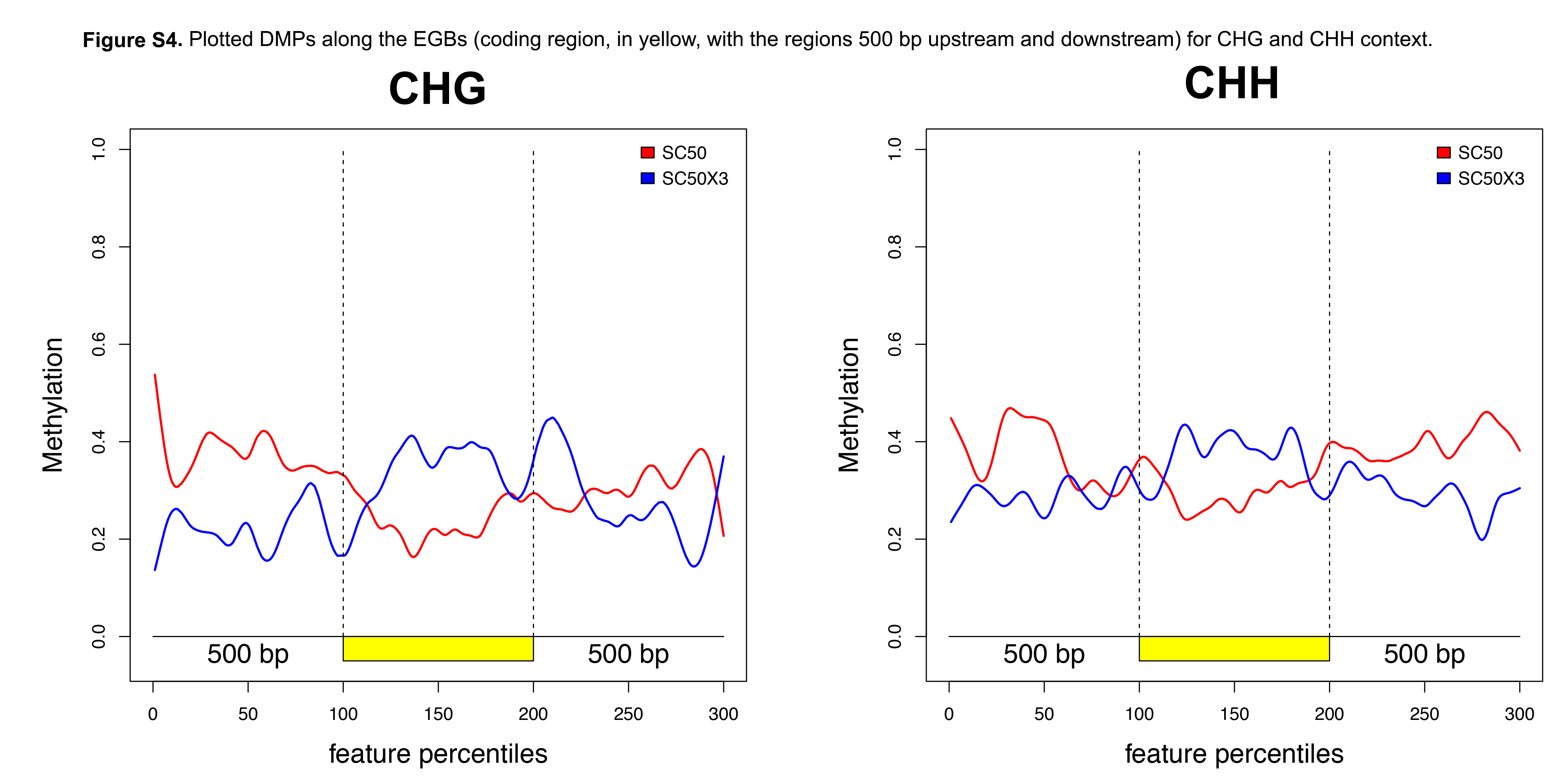

Supplement: Supplementary file 1 [file cells-10-01192-s001.zip › Supplementary files/Figure S4.tif]
